# Pathways to Breast Cancer Recurrence

**DOI:** 10.1155/2013/290568

**Published:** 2013-02-28

**Authors:** Aamir Ahmad

**Affiliations:** Department of Pathology, Karmanos Cancer Institute, Wayne State University School of Medicine, Detroit, MI 48201, USA

## Abstract

Breast cancer remains a deadly disease, even with all the recent technological advancements. Early intervention has made an impact, but an overwhelmingly large number of breast cancer patients still live under the fear of “recurrent” disease. Breast cancer recurrence is clinically a huge problem and one that is largely not well understood. Over the years, a number of factors have been studied with an overarching aim of being able to prognose recurrent disease. This paper attempts to provide an overview of our current knowledge of breast cancer recurrence and its associated challenges. Through a survey of the literature on cancer stem cells (CSCs), epithelial-mesenchymal transition (EMT), various signaling pathways such as Notch/Wnt/hedgehog, and microRNAs (miRNAs), we also examine the hypotheses that are currently under investigation for the prevention of breast cancer recurrence.

## 1. Breast Cancer: The Problem

Breast cancer is a major health problem that affects the lives of millions. For the year 2012, it was estimated that 226,870 women in the United States will be diagnosed with breast cancer and that 39,510 women will succumb to it [1, 2]. With these numbers, breast cancer is the leading cancer diagnosed in the US women and is second only to lung cancer in terms of total fatalities [1]. It is generally recognized that much scientific advancements have been made in the area of breast cancer research, and it is because of these efforts that the chances of disease-free survival of breast cancer survivors have increased tremendously over the last few decades. However, this applies only if the breast cancer is diagnosed at an early stage and is limited to the primary organ. Once breast cancer metastasizes to other organs, the therapeutic options are very limited and the success rate of managing such patients in clinics is dismal. 

The challenges in managing breast cancer patients are very many. First of all, although many risk factors have been associated with the possible initiation and progression of disease, nothing concrete is established that can potentially prevent the primary disease or its progression and metastases. Additionally, there are well-studied disparities in breast cancer that include socioeconomic disparities [3] as well as the racial disparities [4]. All this information seems to suggest that no two women have equal chances of developing the disease. Even when comparing among breast cancer patients, there are not very reliable predictors of aggressiveness. 

The advanced stage breast cancers are broadly characterized by one or both of these—metastases and drug resistance. Metastases to organs such as lungs, bones, brain, and liver severely limit the option of surgical intervention and also suffer from the lack of targeted therapies. Drug resistance is another challenge that is clinically very relevant. Many breast cancer patients do not respond to targeted therapies from the start, and this is called *de novo drug resistance*. A number of breast cancer patients, however, do respond to targeted therapies such as tamoxifen and trastuzumab and show signs of improvement initially, only to turn refractory to these treatments with the passage of time, called *acquired resistance* [5, 6]. Metastases as well as drug resistance phenotypes almost always result in poor prognosis and a disease condition that is progressively aggressive.

**Figure 1:**
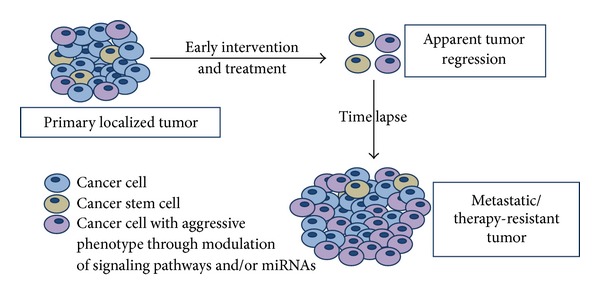
Current understanding of breast tumor recurrence. Breast cancers localized at primary breast location and treated early can still relapse due to (a) existence of cancer stem cells and (b) transformation of cancer cells into a relatively aggressive phenotype. Cancer stem cells as well as transformed cancer cells are resistant to conventional therapies and highly metastatic. Recurrent breast cancers are typically marked by high percentage of aggressive cells.

As mentioned previously, a breast cancer patient has very good chances of a disease-free survival if the cancer is caught and treated early. It is important to mention that the term “early” is very subjective. In many cases, the cancer is thought to have been treated early only to discover its reappearance years after the first intervention (Figure 1). This phenomenon is “tumor recurrence” and the subject of our discussion here. 

## 2. Breast Cancer Recurrence

Recurrence of breast cancer is a major clinical manifestation and represents the principal cause of breast cancer-related deaths [7]. A number of researchers have tried to predict some sort of pattern for breast cancer recurrence. This has included studies in various breast cancer subtypes wherein breast cancers are characterized by the presence of receptors such as estrogen receptor (ER), progesterone receptor (PR), and HER2/ErbB2 receptor (HER2) or by the absence of all of them, the triple negative breast cancers (TNBCs). A differential pattern of recurrence between different breast cancer subtypes has been suggested, and it appears [8–10] that ER-negative breast cancers are associated with higher risk of recurrence during the initial 5 years after diagnosis, compared to ER-positive breast cancers. Thereafter, the risk of recurrence chronically increases in ER-positive breast cancers for the next 10 years, and at 15 years following diagnosis, the risk appears to be equal for both subtypes. In ductal carcinoma in situ, it has been analyzed that the ER-negative/PR-negative but HER2-positive cancers have higher risk of recurrence, compared to ER-positive/PR-positive/HER2-negative cancers [11]. The TNBCs, marked by absence of ER/PR/HER2, are generally associated with high risk of recurrence with particularly high risk of distant recurrences in brain and visceral metastases, compared to receptor positive tumors [12]. 

In addition to the simple classification of breast cancers described previously, there are other subclassifications of breast cancers as well, such as the one that classifies breast cancers into luminal A, luminal B, basal, and HER2 enriched [13]. The luminal A subtype includes ER-positive and/or PR-positive, HER2-negative breast cancers; luminal B subtype includes ER-positive and/or PR-positive, HER2-positive breast cancers; basal subtype includes ER-negative, PR-negative, HER2-negative breast cancers which may also be positive for EGFR, and HER2-enriched subtype includes ER-negative, PR-negative, and HER2-overexpressing breast cancers. The existence of such subtypes, which are at time overlapping but most of the time so distinct, presents a challenge to the choice of appropriate therapy. This has led to the proposal of personalized therapy that fits the needs of individual patients.

Irrespective of the underlying breast cancer subtype, a large number of advanced stage breast cancers are marked by metastases to lymph nodes, and, overall, the presence of axillary lymph node metastases is associated with considerable poor disease-free as well as overall survival [14]. Axillary lymph node metastases remain a very important prognostic variable, and identification of molecular markers for development of lymph node metastases can potentially help intervene early reducing the chances of breast cancer recurrence [15]. 

## 3. Cancer Recurrence in Breast Cancer Patients Undergoing Surgery

Surgical intervention is one of the options for breast cancer patients [16]. The choice for surgical intervention largely depends on the cancer stage. For patients presenting with early stage breast cancer, the two most common treatment options available are breast conserving surgery followed by radiation therapy or mastectomy [17–19]. Mastectomy usually does not need subsequent radiation therapy. It seems that there are distinct risk factors of recurrence in breast cancer patients undergoing mastectomy versus those choosing breast conserving therapy [20]. Whereas lymph node involvement and tumor size are major risk factors after mastectomy, the young age and presence of ductal carcinoma in situ are major risk factors after breast conservation therapy. The distinct clinical and histopathological determinants result in differential response to radiotherapy and point to the need for more robust personalized therapies and follow-up procedures for patients opting for different surgical interventions. Further complicating the decision of surgeons performing breast conserving therapy is the realization that surgical margins also seem to impact the recurrence of breast cancer [21]. In a study [17] that scanned published reports and systematically reviewed the local recurrence in breast cancer patients undergoing mastectomy with or without breast reconstruction, it was concluded that the recurrence rates were not higher in patients that underwent breast reconstruction. Also, radiation therapy is generally associated with reduced recurrence in breast cancer, and a recent meta-analysis [22] of 10,801 patients from 17 randomized trials has concluded that radiotherapy reduces the recurrence rate by half and the death rate by about a sixth in patients that have undergone breast conserving surgery. Interestingly, this study also identified many factors that might influence the extent of radiotherapy-induced benefits. Such factors include age, cancer grade, estrogen receptor status, and use of tamoxifen as well as the extent of surgery. 

In a study [23] that looked at the influence of molecular subtypes of breast cancers, namely, ER-positive, PR-positive, and HER2-overexpressing as well as TNBC in relation to recurrence in patients with mastectomy versus breast conserving surgery, a differential rate of recurrence was observed in patients with different molecular subtypes. This study analyzed data from 15 different studies covering a total of 12,592 patients of which 57% underwent breast conserving therapy while 43% underwent mastectomy. It was observed that among the patients that underwent breast conserving therapy, patients with ER-positive and PR-positive breast cancers had many reduced instances of recurrence than HER2-overexpressing and TNBC patients. Similar results were observed for mastectomy patients as well where ER-positive and PR-positive patients were again found to be at a lesser risk of recurrence compared to HER2-overexpressing and TNBC patients. Although both HER2-overexpressing and TNBC patients were found to be at a higher risk of recurrence, a direct comparison between the two subtypes revealed that HER2-overexpressing breast cancer patients presented higher risk of recurrence in patients undergoing breast conserving surgery. In the mastectomy patients, the risks of recurrence in HER2-overexpresing and TNBC patients was not found to be statistically different. This analysis identified a differential risk of breast cancer recurrence in patients with different molecular subtypes and underlines the need to consider breast cancer subtype while managing breast cancer patients for disease-free survival.

In the context of ipsilateral breast tumor recurrences after breast conserving surgery, two interesting hypotheses have been proposed [24] which seek to better define our understanding of true tumor recurrences. First of all, it has been proposed that the local recurrences might actually initiate long before the diagnosis of primary tumor and may be recorded as multifocal primary tumor at the time of diagnosis, and, secondly, true local recurrences might actually never metastasize to distant organs. Breast cancer was proposed as an ideal cancer for such studies because of the availability of enormous clinical data for analysis. Another interesting theory for cancer recurrence is the wound-oncogene-wound healing (WOWH) hypothesis [25] which is based on the observed interrelationships between precancerous lesions, cancer, oncogenes, wound healing, and cancer recurrence. The essence of this theory is that the “wounds,” exemplified by physical (such as radiations), chemical (such as carcinogens) and biological (such as inflammation, aging, and reactive oxygen species) damages, trigger the oncogenes to produce cytokines resulting in recruitment of stem cells and tissue remodeling. All this leads to generation of cancer mass, particularly with continued existence of wounds, and ultimately results in death of the organism.

## 4. Molecular Factors That Influence Breast Cancer Recurrence

It is evident that breast cancer is particularly lethal when it recurs. The causes for such breast cancer recurrence remain completely unknown, except for many putative molecular markers that are under active investigation for their possible role in determining the recurrence. This section discusses many such factors with emphasis on studies that have linked individual molecules/signaling factors with breast cancer recurrence and/or disease-free survival.

### 4.1. Cancer Stem Cells

Over the last few decades a number of hypotheses have been proposed to explain tumor recurrence such as clonal selection, angiogenic dormancy, and, more recently, CSCs [26, 27]. CSCs are cells within populations of cancer cells or tumors which possess the capacity to self-renew and produce heterogeneous lineages of cancer cells [28]. CSCs, by virtue of being stem cells, have tumor-initiating capabilities. CSCs are now believed to persist in tumors as distinct populations that are fundamentally associated with drug resistance, tumor recurrence, and metastasis. Our inability to prevent tumor recurrence in clinics points to the fact that the knowledge of underlying factors is not yet mature enough, and much more needs to be done. While CSC theory appears to be attractive, it has its own pitfalls [29]. A number of molecular pathways have been proposed to play a role in maintenance of CSC phenotype which is further complicated by the observation that none of the molecular markers of CSCs seems to be universally relevant. Most of the research is cancer specific, and the factors/pathways relevant in one cancer may or may not be relevant targets for therapy in other cancers. 

**Figure 2:**
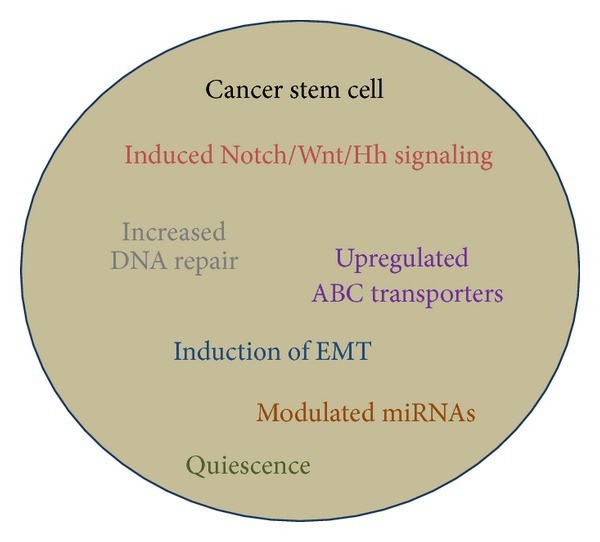
Multiple factors that define the “stemness” in cancer stem cells.

The majority of research investigations on the role of CSCs in tumor recurrence have focused on their role in the context of resistance against chemotherapy; however, it is increasingly being advocated that CSCs determine the resistance to radiation therapy as well. When tumors are subjected to radiation therapy as part of the anticancer therapy, CSCs still thrive, and a higher proportion of CSCs correlates with increased resistance to radiation therapy [30]. Multiple signaling pathways in breast CSCs determine their resistance to radiations, such as heat shock protein 27 (hsp27), EMT, and NF-*κ*B signaling [31]. A number of mechanisms are believed to contribute to the CSCs-induced resistance to drugs and tumor recurrence, and these include quiescence, upregulation of ABC transporters, highly efficient DNA repair systems, and upregulation of several signaling pathways [32] (Figure 2). Quiescence is the state of temporary inactivity. The role of quiescence in CSCs activity has its basis in the observation that a number of chemotherapeutic regimes target rapidly proliferating cancer cells. Thus, CSCs, through their ability to proliferate slowly with intermittent phases of quiescence, are able to evade the cytotoxic effects of anticancer drugs. The role of ABC transporters in drug resistance of cancer cells has long been advocated [33, 34]. Drug-resistance mechanisms can either interfere with the delivery of drugs to tumor cells or arise within the cancer cells, such as ABC transporters, leading to alterations in drug sensitivity. ABC transporters increase the drug efflux from tumor cells leading to reduced intracellular drug concentrations which are not cytotoxic enough. Drug resistance mediated by ABC transporters is believed to mediate resistance against the entire class of drugs as opposed to being drug specific. This is why resistance against anticancer drugs, paclitaxel, doxorubicin, or vinblastine, is frequently due to increased expression of ABC transporters [33, 35]. ABC transporters are upregulated in hematopoietic stem cells supporting their role in conferring stem cell-like phenotype [34, 36–38] and the drug resistance of CSCs [39, 40]. 

During the last several years, a role of CSCs in tumor progression, metastasis, and angiogenesis has been established [41–44]. Breast cancer is in the forefront of ongoing studies on the role of CSCs in mediating metastasis as well as resistance to current pharmaceutical regimes, and this is believed to involve a complex interplay of several cell types, cytokines, cell growths, and signaling pathways [45, 46]. Also, a number of cell surface markers such CD44 (high)/CD24 (low)/ALDH-positive have been associated with the CSCs [47]. A number of putative molecular markers of CSCs are being pursued for in-depth clinical studies [48, 49]. The phenomena of drug resistance and tumor recurrence are intricately related because, in order to recur, cancer cells need to overcome the cytotoxic effects of drugs that are used to control the growth of these cancers in clinics. Thus, drug resistance, mediated by CSCs, goes hand in hand with the tumor recurrence [50].

### 4.2. Epithelial-Mesenchymal Transition

The process of EMT has attracted much interest in recent years with regard to breast cancer aggression. Its association with CSC phenotype further underscores its importance in recurrence of breast tumors [51–53]. Progression of most carcinomas towards malignancy is associated with the loss of epithelial differentiation and a switch toward mesenchymal phenotype, which is accompanied by increased cell motility and invasion. The process of EMT, by which epithelial cells undergo remarkable morphological changes is characterized by a transition from epithelial cobblestone phenotype to elongated fibroblastic phenotype. This process involves loss of epithelial cell-cell junction, actin cytoskeleton reorganization, and upregulation of mesenchymal molecular markers such as vimentin, ZEB-1, ZEB-2, fibronectin, and N-cadherin [54]. A disassembly of cell-cell junction, including downregulation and relocation of E-cadherin and zonula occludens-1 as well as downregulation and translocation of *β*-catenin from cell membrane to nucleus, is known to be the mechanism for the induction of EMT [55]. Epithelial cells have a regular cell-cell junction and adhesion which inhibits cell movement of individual cells. In contrast, mesenchymal cells have weaker adhesion between cells compared to their epithelial counterparts, which renders mesenchymal cells more motile functions and confers more invasive characteristics. In addition to classical markers of EMT, such as E-cadherin, vimentin, and ZEB-1/ZEB-2, the process of EMT is also influenced by several other signaling molecules, particularly those from Notch and Wnt signaling pathways.

Since a switch from epithelial to mesenchymal phenotype is a good indicator of aggressiveness of cancer cells, it is desirable for an anticancer agent to reverse this phenomenon; that is, revert back from mesenchymal to epithelial phenotype (MET) [56]. An upregulation of epithelial markers and/or downregulation of mesenchymal markers is considered a reliable indication of the ability of any therapeutic agent to reverse EMT, thereby reducing the invasion and metastasis of cancer cells [57]. In an early report on the subject, snail expression was shown to induce EMT which also correlated with levels in recurrent tumors *in vivo* [7]. Mechanistically, it was shown that snail was sufficient to not only induce EMT in primary tumor cells, its induced levels predicted reduced relapse-free survival and promoted breast tumor recurrence *in vivo*. A review of public databases of breast cancer further supported such role of snail1 in breast cancer recurrence [58]. Another study on breast cancer cohort also supported a link between snail, twist, and significantly reduced relapse-free survival [59]. Since snail and twist regulate E-cadherin and N-cadherin expression, the recurrent tumors also expressed reduced E-cadherin and increased N-cadherin. It has been suggested that whereas snail activity is required for EMT initiation, twist plays a role in the maintenance of EMT [60]. Such interplay and spatial cooperation of these factors are crucial to the process of breast cancer metastasis and predictive of tumor recurrence. The role of twist and N-cadherin in EMT of breast cancer cells has been demonstrated in other investigations as well [61].

The role of TGF-*β* in EMT of breast cancer is well understood [62–65]. In a study suggestive of adverse effects of anticancer drugs, doxorubicin was demonstrated to induce TGF-*β*-driven EMT that could result in CSCs and the resistance to chemotherapy [66]. This is another indication of a complex relationship between EMT, CSCs, drug resistance, and the breast cancer recurrence. The TGF-*β*-induced EMT and the resulting CSC phenotype have also been characterized as the ones with low claudins [67]. Such claudin-low phenotype is marked by increased resistance to oxaliplatin, etoposide and paclitaxel. A link between cell cycle regulatory cyclin D1 and EMT has also been reported [68]. A recent study [69] evaluated the correlation, if any, between EMT markers and poor clinical outcome. The EMT markers chosen were E-cadherin, vimentin, and slug. Immunohistochemical analyses were done on samples from 441 patients, and the patients were followed up for data on survival. A high expression of mesenchymal marker slug and a lower expression of epithelial marker were observed to correlate with tumor grade, presence of lymph node metastases and advanced stage breast cancer. The follow-up survival data clearly linked low E-cadherin and high vimentin/slug to poor prognosis and tumor recurrence. 

Our work with autocrine motility factor (AMF) in the metastasis of breast cancer cells has highlighted an important mechanistic involvement of EMT [70]. AMF, also known as phosphoglucose isomerase, has been linked to poor patient survival and tumor recurrence [71]. An earlier report suggested a downregulation of E-cadherin in AMF-expressing cells and, conversely, its upregulation in AMF-silenced cells which was consistent with EMT [72]. We observed that the EMT induction in AMF-expressing cells was marked by not only a downregulation of epithelial marker E-cadherin but also by the induction of mesenchymal markers vimentin, ZEB-1, and ZEB-2, and all this involved a mechanistic involvement of miRNAs [70]. The process of EMT is evidently complex, which often involves multiple factors—there are multiple determinants/molecular markers of epithelial versus mesenchymal phenotype, all of which might not be found to be altered under all experimental conditions. Also, the markers of EMT are interregulated as well as regulated by other factors such as miRNAs [73]. There are many levels of regulation, the details of which are only beginning to emerge.  Table 1 lists the various molecular markers of EMT that have been associated with breast cancer recurrence.

### 4.3. *β*1-Integrin

The recognition of *β*1-integrin in recurrence of breast cancer originates from the observation that a majority of cancers, including breast cancer, enter a state of dormancy. Tumor dormancy is the stage where cancer cells, after primary cancer intervention and apparent treatment, enter a state wherein they virtually go undetected waiting for right time and conditions to trigger cancer recurrence [8]. Clinical proof for existence of tumor dormancy was provided in a study that looked for circulating tumor cells in 36 dormancy patients [74]. Of these 36 patients, 13 had circulating tumor cells 7 to 22 years after mastectomy but the patients had no evidence of clinical disease suggesting that tumor cells do undergo dormancy and they remain in systemic circulation. Incidentally, circulating tumor cells have themselves been proposed as molecular determinants of breast cancer recurrence [75]. The dormant cells are refractory to chemotherapy as well as radiation therapy [76, 77]. A major challenge in our understanding of this whole process is the elucidation of mechanism(s) by which the dormant tumor cells eventually decide to leave dormancy and form tumors at distant sites leading to recurrence of disease many years after the initial “successful” treatment of breast cancer. *β*1-integrin is one molecular factor that has been proposed to play an important role in the switch from dormant state to that of metastatic progression in different human cancers [78], including breast cancer [79, 80]. These mechanistic investigations revealed interactions of *β*1-integrin with several factors such as focal adhesion kinase (FAK), urokinase-type plasminogen activator receptor (uPAR), extracellular signal-regulated kinase (ERK), and epidermal growth factor receptor (EGFR), all of which influence tumor microenvironment and have been implicated in breast cancer progression [81–88]. It is, therefore, not surprising that the inhibitors of *β*1-integrin have shown promise in clinical trials for prevention of breast cancer metastasis and recurrence [78].

### 4.4. Notch Signaling

Notch signaling is associated with normal developmental processes [89, 90], and the loss of Notch signaling leads to embryonic lethality [91]. Activation of Notch signaling is believed to be a “hallmark” of aggressive cancers. There are four known Notch-family receptors in humans: Notch-1, Notch-2, Notch-3, and Notch-4. The ligands for Notch receptors identified in mammalian cells are Jagged-1, Jagged-2, Delta-like 1, Delta-like 3 and Delta-like 4. Binding of Notch ligands to their receptors initiates a proteolytic cascade resulting in the release of intracellular part of Notch which translocates to nucleus and regulates the transcription of several target genes [92]. 

The ligand Jagged-1 was evaluated as a determinant of breast tumor recurrence in a study that examined 887 samples from a prospectively accrued cohort [93]. Reduced disease-free survival was used as a criterion to propose the role of Jagged-1 as a factor for breast tumor recurrence. It was observed that the tumors expressing high levels of Jagged-1 mRNA and protein correlated with reduced disease-free survival. Interestingly, mRNA levels of Jagged-1 alone were not a very reliable factor in determining poor disease-free survival, particularly when adjusted for the presence/absence of other breast cancer biomarkers. These observations suggested a functional role of active Jagged-1 in determining the aggressive phenotype of recurrent breast cancers. A correlation of Jagged-1 with ER/PR negativity was also observed which was further confirmed by another study [94] which reported higher Jagged-1 levels in triple negative breast cancer cells, compared to ER-positive and HER2-expressing cells. This latter study [94] took a genomics approach to list downstream targets of Jagged-1 and focused on cell cycle regulatory factor cyclin D1 which was found to be a direct target of Notch-family proteins Notch-1 and Notch-3 as well. Both Jagged-1 and cyclin D1 levels correlated well with triple negative breast cancers, and Jagged-1 was found to play an important role in binding of Notch-1 to cyclin D1 promoter, an event necessary for cell cycle progression. 

As discussed earlier in this paper, there is a direct connection between CSCs and tumor recurrence. In a study that was designed to investigate the role of Notch family in stem-cell-like phenotype [95], the levels of Notch family receptors were determined in breast cells that were enriched for breast CSC markers ESA and CD44. In these cells with enriched CSC markers, Notch-1 signaling activity was found to be increased 4-fold while the Notch-4 signaling activity was found to be even higher with 8-fold induction. Mechanistic studies were carried out through downregulation of Notch-1/Notch-4, and it was reported that Notch-4 inhibition was more robustly associated with downregulation of stem-cell phenotype. This and another *in vitro* study [96] suggested a role of Notch signaling in breast CSCs. A role of Notch-3 in CSC quiescence has also been suggested [97] which might be relevant to tumor recurrence because, as discussed previously, quiescence happens to be one of the mechanisms through which CSCs evade the cytotoxic effects of anticancer drugs and radiation therapy. In a mouse model of HER2 breast cancers [98], inhibition of Notch signaling through the use of a *γ*-secretase inhibitor MRK-003 was found to kill CSCs *in vitro* as well as *in vivo*. This indicates that Notch signaling is relevant not only to triple negative breast cancers, but also to HER2-over-expressing breast cancers. Recently, it has been reported that monoclonal antibodies against Notch-1 have remarkable activity against CSCs [99] which clearly demonstrates a correlation between Notch signaling and the breast tumor recurrence through CSC phenotype.

In addition to their role in breast tumor recurrence through regulation of CSCs, Notch-family members have also been linked to uPA signaling which further supports their ability to influence tumor recurrence [100]. uPA signaling is elevated in several human cancers [82], and an inhibition of uPA and its receptor uPAR has been demonstrated by us to result in reduced cell proliferation and invasion of breast cancer cells [83]. The study by Shimizu et al. [100] confirmed an association between Jagged-1 and uPA in the basal type breast cancer subtype. Activation of Notch-1 by Jagged-1 was found to result in upregulation of uPA signaling which resulted in breast cancer progression.

Tumor recurrence is closely related to drug resistance. In a study that explored the possibility of inhibiting Notch signaling to overcome trastuzumab resistance [101], it was found that combined inhibition of Notch and HER2 signaling pathways can indeed decrease recurrence rates for breast cancers that are characterized by HER2 overexpression. HER2 is overexpressed or amplified in 20%–30% invasive breast cancers [6], and HER2-overexpressing breast cancers are invariably linked to worse prognosis and poor survival [102]. In normal tissues, expression of HER2 is low but in breast cancer cells its expression is so high that there can be up to two million receptors on a single cell [102–104]. Breast cancer cells, marked by overexpression of HER2, can be effectively targeted by HER2-specific therapies. Marketed as “Herceptin,” trastuzumab is a monoclonal antibody that is very effective in HER2-overexpressing breast cancers. Trastuzumab has unique place in cancer research as this was the first monoclonal antibody approved in 1998 for use against a solid tumor. Trastuzumab remains a standard of care for the treatment of HER2-overexpressing breast cancers in adjuvant as well as in metastatic recurrent breast cancer settings. However, trastuzumab therapy also suffers from development of drug resistance, that is, resistance against trastuzumab [6]. In this regard, the study by Pandya et al. [101] was assumed to be significant because it suggested that targeted inhibition of Notch signaling might benefit patients whose tumors have turned resistant to trastuzumab. This study compared trastuzumab-sensitive HER2-overexpressing BT474 breast cancer cells with their trastuzumab-resistant counterparts. *γ*-secretase inhibitors LY411575 and MRK-003 were used to inhibit Notch signaling.

Collectively, these studies seem to indicate that selective targeting of Notch signaling can reduce breast cancer tumor recurrence and increase disease-free survival, an idea that needs to be further investigated [105, 106].

### 4.5. Wnt Signaling

Wnt family of proteins includes several glycoproteins that activate various intracellular pathways after binding to transmembrane frizzled receptor family proteins or to a complex comprising of frizzled and LDL receptor-related proteins 5/6. The best studied Wnt pathway, the Wnt/*β*-catenin pathway, is known as the “canonical” Wnt pathway. In the absence of Wnt ligands, *β*-catenin is recruited into a destruction complex of adenomatous polyposis coli and axin, which induce the phosphorylation of *β*-catenin through casein kinase and glycogen synthase kinase 3 (GSK3). This leads to ubiquitylation and proteasomal degradation of *β*-catenin. When Wnt proteins bind to frizzled, disheveled is activated which recruits the destruction complex to plasma membrane, inhibiting GSK3 and thus preventing phosphorylation of *β*-catenin. *β*-catenin then accumulates in the cytoplasm and translocates to the nucleus, where it activates target genes [107].

Similar to Notch signaling, a role of Wnt signaling in breast tumor recurrence has also been suggested [44, 106, 108]. A direct proof was provided through a study on tumor suppressor Wnt-5a [109]. It was found that the expression of Wnt-5a was lost in 78% of the patients with recurrent disease, compared with only 35% of recurrence-free patients. This study suggested the usefulness of Wnt-family member Wnt-5a as a possible biomarker for breast tumor recurrence with a negative correlation. Loss of Wnt-5a significantly increased metastases but not the proliferation of breast cancer cells. A follow-up study by the same research group [110] came up with the minimal amino acid sequence that could mimic Wnt-5a effects. Further evidence in support of a role of Wnt signaling in recurrent and invasive breast cancers came from the study on HBP1, the transcriptional repressor of Wnt signaling [111]. This study identified a correlation between inactivating HBP1 mutations and invasive breast cancers in clinical breast cancer samples. The mutations in HBP1 rendered them ineffective in suppressing Wnt which results in increased invasive potential, a hypothesis that was also tested by using RNA interference to reduce HBP1 levels. Statistical analysis on breast cancer patient database revealed a direct relationship between reduced HBP1 levels (or increased Wnt signaling) and increased cancer recurrence. Debies et al. [112] provided a direct mechanism of activated Wnt signaling that resulted in breast cancer recurrence after targeted therapy. 

In a study that looked at Wnt signaling with relation to tumor relapse in various subtypes, it was determined that Wnt signaling was upregulated in the basal subtype, particularly in brain-specific relapse [113]. In the triple negative breast cancer model represented by CRL-2335 and MDA-MB-468 cells, inhibition of Wnt signaling by a treatment regime comprising of cisplatin plus TRAIL was found to be most effective in inhibiting the CSCs [114]. A number of treatments were compared in this study, namely, PARP inhibitors, paclitaxel, docetaxel, cisplatin, and cisplatin plus TRAIL. The involvement of Wnt signaling in the most effective treatment suggests its importance in CSC phenotype that is responsible for breast tumor recurrence. Thus, it appears that Wnt signaling contributes to CSC phenotype and, additionally, is transcriptionally important for the determination of aggressive breast tumors that recur and relapse. A recent report has suggested that Wnt signaling is commonly activated in metaplastic breast carcinomas [115]. Although these metaplastic carcinomas represent only 1% of total invasive breast carcinomas, they have a particular high risk of local recurrence.

### 4.6. Hedgehog Signaling

Hedgehog (Hh) pathway is mainly involved in the development of organs in most animals [116]. Hh gene was first identified in *Drosophila*, and the three mammalian counterparts, Sonic Hedgehog (Shh), Desert Hedgehog (Dhh), and Indian Hedgehog (Ihh), were identified later [117]. Shh binds to its 12-pass transmembrane receptor, Patched, resulting in derepression of Smoothened [116–118] and leading to the activation of Gli2 in the cytoplasm. Gli2 translocates to the nucleus and regulates the transcription of Shh-pathway target genes, including Gli1 and Patched1. 

Similar to Notch and Wnt signaling pathways discussed previously, there is evidence to suggest a correlation between Hh signaling and tumor recurrence [108, 119, 120]. For example, in a study [121] that evaluated 279 patients with invasive breast ductal carcinoma, expression of Hh was found to be a strong determinant of increased risk of metastases and breast-cancer-specific deaths, all hallmarks of tumor recurrence and lethal disease. High epithelial Hh ligand and high stromal Gli1 were observed to be crucial predictors of overall survival. The ligand Shh has also been shown to increase vascularity and angiogenesis in a novel VEGF-independent pathway *in vitro* as well as in clinical samples from breast cancer patients [122]. Angiogenesis and increased vascularity play an important role in cancer metastases, and bone metastases are very common in advanced breast cancer patients. There is evidence to suggest a role of Hh signaling in altering the bone microenvironment making it suitable for homing of breast cancer cells [123]. It has therefore been suggested that selective targeting of Hh signaling might be beneficial in preventing bone metastases of breast cancers [124, 125].

As with other potential biomarkers of tumor recurrence discussed previously, there has been an interest in evaluating Hh signaling members in different breast cancer subtypes as well. In one such study [126], it was observed that Gli1 expression correlated well with basal breast cancer subtype, and the patients with high nuclear expression of Gli1 had significantly reduced survival time. In another study that focused on triple negative breast cancers [127], it was observed that Smoothened and Gli1 were significantly upregulated in triple negative breast cancers, as compared to the other breast cancer subtypes. The individual members of hedgehog signaling, Shh, Gli1, and Smoothened, were found to correlate with increased metastasis and tumor grade while, at the same time, Gli1 expression was inversely associated with ER. This histological evidence supports a role of Hh signaling in advanced breast tumors that relapse. In the context of drug resistance and breast tumor recurrence, Smoothened and Gli1 were reported to be significantly upregulated in tamoxifen-resistant derivative of otherwise tamoxifen-sensitive MCF-7 and T47D breast cancer cells [128]. This study also established a link between PI3K/Akt signaling pathway and the Hh signaling in the development of tamoxifen-resistant breast cancers. 

### 4.7. miRNAs

MicroRNAs (miRNAs) are small (19–24 nucleotides) noncoding RNA molecules which down-regulate gene expression by interacting with sequences located in the 3′ untranslated region (UTR) of target mRNAs, resulting in either translational repression or degradation of mRNAs [129]. Regulation of oncogenes/tumor suppressor genes by miRNAs is now recognized as a key step in the progression of human malignancies [130], and it is dependent on sequence complementarities. miRNAs largely function via repression of their target genes; therefore, if the target gene of a miRNA is an oncogene, that particular miRNA will be tumor suppressive. In contrast, an oncogenic miRNA is the one whose target is a tumor suppressor gene [131]. miRNAs represent attractive targets for therapy, particularly in breast cancer, and breast cancer is a very well-studied cancer with regard to evaluation of various microRNAs that might play key roles in its progression [132–135]. Multiple reports have shown that the loss and gain of specific miRNAs influence the processes of invasion and metastasis of human breast cancers [73, 136–138]. 

miR-126 and miR-335 were the early miRNAs connected with breast cancer recurrence when it was proposed that the expression of these two miRNAs is lost in a majority of breast cancer patients who suffer relapse [139]. Consequently, loss of both of these miRNAs is associated with poor metastasis-free survival. This was followed by the suggestion that miR-31 and miR-34c might be the miRNAs that are upregulated in recurrent breast tumors [140]. A subsequent study confirmed the downregulation of miR-335 in recurrent tumors, and this miRNA was identified as a robust inhibitor of tumor reinitiation [141]. In a study that employed global miRNA together with mRNA expression profiling to list miRNAs that might have a prognostic value in determining distant relapse-free survival, several miRNAs were found to be prognostically important in ER-positive as well as ER-negative breast cancers [142]. When matched for the mRNA targets, the two miRNAs that stood out for their putative prognostic value were miR-210 and miR-128a. More recent data has connected miR-92a with better outcome and reduced tumor recurrence [143], and miR-9 with increased tumor recurrence [144]. An interesting observation was made in a study on miR-34a when its expression was examined in 1172 breast tumors [145]. First of all, it was detectable in most of the samples studied. The tumors with high expression of miR-34a represented aggressive breast cancers but the tumors with lower expression suffered from significantly increased tumor recurrence. Thus, this miRNA presented a novel and peculiar finding which needs to be explored further. Another miRNA down-regulated in recurrent breast tumors, miR-320, is believed to function through its regulation of phosphatase and tensin homolog (PTEN) [146]. 

As discussed before, breast cancer frequently metastasizes to bone, and that metastasis is one of the primary reasons for tumor recurrence. Several investigations have been carried out to uncover the miRNA regulation of breast cancer metastases that might be the reason for tumor relapse. In one such study that focused on bone metastasis of breast cancer, miR-21 and miR-181a were found to be enriched in bone metastatic breast cancers leading to poor prognosis [147]. This study primarily utilized miRNA microarray to distinguish the miRNA signature between 4 patients who represented recurrent breast cancer versus 4 breast cancer patients without recurrence. Later, the results were validated in bone marrow samples from 291 additional patients.

In the context of drug resistance and breast cancer recurrence, Bergamaschi and Katzenellenbogen [148] studied the tamoxifen-induced induction of 14-3-3*ζ* which confers tamoxifen resistance leading to cancer relapse. The study revealed a mechanistic role of miR-451 which was down-regulated by tamoxifen leading to derepression of its target 14-3-3*ζ*. The tamoxifen-resistant cells were marked by upregulated 14-3-3*ζ* and down-regulated miR-451. A more recent study has indicated that there is no single miRNA profile predictive of outcome following tamoxifen treatment [149]. This points to the limitations and challenges in the field of miRNA research and the need for more robust investigations. 

EMT and its regulation by miRNAs are not novel information anymore. However, a recent study evaluated this connection with possible clinical relevance. This study found a regulation of mesenchymal marker vimentin by miR-30a [150]. Since vimentin is associated with EMT and an invasive phenotype, miR-30a correlated with reduced invasion and breast cancer aggressiveness. The clinical importance of this regulation was revealed with the observation that breast cancer patients with reduced levels of miR-30a had poor prognosis, increased metastases and worse prognosis. This study provided further evidence connecting EMT with increased breast cancer recurrence.

Detection of miRNAs in circulation is a hot topic attracting attention in an attempt to predict the outcome of therapeutic regime as well as chances of tumor relapse. In one such recent study [151], more than 800 miRNAs were actually detected in the serum of breast cancer patients. After vigorous analyses, miR-122 stood out as the miRNA that was significantly induced in metastatic breast cancer patients with recurrence. Circulating miRNAs have also been evaluated in relation to resistance to chemotherapy, and miR-125b expression in circulation has been linked to increased chemoresistance [152]. This knowledge holds a lot of promise particularly for the early stage patients who can be monitored on a regular basis for the possible chances of cancer relapse which can then be handled clinically at an early stage. Although a significant advancement, use of circulatory miRNAs in clinical prognosis is still in its early stages which needs to be developed further [153].

The last few years have seen an exponential increase in the number of investigations focused on the functionality of miRNAs in breast cancer progression. There has also been an interest in studying miRNAs with possible implications in predicting breast cancer recurrence.  Table 2 lists various miRNAs that have been shown to relate to recurrence of breast cancer. The area of research involving miRNAs is relatively new, but has captured the imagination of a large number of researchers. More robust investigations are needed to further exploit the potential of these tiny regulatory molecules.

## 5. Therapeutic Options to Prevent Breast Cancer Recurrence

The preceding section focused on many factors that are being pursued in an attempt to better understand breast cancer recurrence. The ultimate goal of such knowledge will be to utilize it for the development of targeted therapies to specifically prevent relapse of breast cancer. Here is a list of options that are already under investigation(s). The list is not very exhaustive and is purposely kept so, to reflect on the limited options that are available, so as also to avoid being overly enthusiastic about the progress being made in the field.

### 5.1. Aromatase Inhibitors

As mentioned earlier in this paper, a subset of breast cancers are marked by expression of estrogen receptor, and such ER-positive breast cancers require estrogen for their growth and proliferation. MCF-7 cell line developed at our Institute (erstwhile called Michigan Cancer Foundation, MCF), the most widely studied breast cancer cell line in preclinical studies worldwide, represents ER-positive breast cancers. With an importance of estrogen in the sustenance of this breast cancer subtype, it becomes logical to block the production of estrogen in affected breast cancer patients. This can be accomplished by the inhibition of enzyme aromatase that is involved in the production of estrogen. As such, aromatase inhibitors represent a class of compounds/anticancer drugs that can be used for their beneficial effects in ER-positive breast cancer patients [154, 155]. The common aromatase inhibitors in use include those that permanently inhibit aromatase as well as those whose action is reversible. Interestingly, natural anticancer agents such as resveratrol with documented anticancer property [156, 157] are natural aromatase inhibitors [158]. 

Metastases to distant organs, in the initial 2 years following surgery, are the major reasons for tumor recurrences in breast cancer patients treated with tamoxifen, the targeted drug against estrogen receptors [159]. Data from clinical studies have suggested a usefulness of aromatase inhibitors in preventing early stage distant metastases, and, therefore, it has been proposed that initial therapy involving adjuvant use of aromatase inhibitors can be beneficial against tumor recurrence [160, 161]. For many years, a five-year administration of tamoxifen has been the gold standard for endocrine therapy in ER-responsive breast cancer patients, and this notion is increasingly being challenged with the inclusion of aromatase inhibitors in the initial years of therapy [162].

As with most therapies, the use of aromatase inhibitors poses its own challenges, mainly in the form of several possible side effects and complications which need to be discussed upfront [163]. In particular, aromatase inhibitors affect cardiovascular and bone health which needs to be monitored throughout the course of therapy. Estrogen action is important for normal bone mineral density, and, therefore, use of aromatase inhibitors can severely compromise bone mineral density in patients. Bone fracture risk assessment should be an important component of care for patients receiving aromatase inhibitors [164]. Therefore, aromatase inhibitors should be considered for use only in ER-positive patients with high risk of recurrence as opposed to the postmenopausal patients with relatively lower risks of recurrent metastatic disease [165]. The apparent toxicity of aromatase inhibitors prompted Amir et al. [166] to evaluate the existence of improved disease-free survival without any significant overall survival. In this systemic review of seven trials with 30,023 patients, it was found that a 5-year use of aromatase inhibitors was associated with actual increased odds of death that were nonsignificant. Such 5-year use of aromatase inhibitors correlated with reduced recurrence, compared to 5 years of tamoxifen administration or the switching from tamoxifen to aromatase inhibitors. The analyses suggested that cumulative toxicity of aromatase inhibitors might be relevant to lack of overall survival, and the switch therapy involving administration of tamoxifen for 2-3 years followed by a switch to aromatase inhibitors for subsequent 2-3 years might be a more rational and balanced approach.

### 5.2. Bisphosphonates

Bone represents a major site of metastasis in breast cancer patients. Metastases to bone disrupt the normal bone homeostasis resulting in loss of bone integrity and function. In addition to direct metastasis of cancer to bone, several other factors also influence bone integrity, and these include chemotherapy-induced effects as well as the consequences of aromatase inhibitors use [167, 168]. It has been suggested that the breast cancer patients are at particular risk of bone fracture because of administration of aromatase inhibitors [168]. This means that the health of skeletal system of breast cancer survivors is a unique challenge that needs attention. Towards this end, the use of bisphosphonates to prevent bone-metastases-related effects has been suggested, and it is believed that approximately half of the patients taking bisphosphonates report significant improvement in pain [167, 169]. This has resulted in a recommendation that breast cancer patients with confirmed bone metastases should start receiving bisphosphonates right from the time of diagnosis [170].

Bisphosphonates improve the quality of life of bone metastatic breast cancer patients dealing with underlying pain but there is also evidence to suggest that early administration of bisphosphonates can actually help reduce the instance of metastasis to bone [171]. Several preclinical as well as clinical studies have established the ability of bisphosphonates to prevent metastases of breast cancer [172–175]. The use of bisphosphonates has been shown to reduce proliferation, migration, invasion, and metastasis of breast cancer cells in many of these studies. As a direct proof in support of the ability of bisphosphonates to reduce breast cancer recurrence, several clinical trials have demonstrated such ability of bisphosphonates clodronate and zoledronic acid [176]. A number of studies support an association between bisphosphonates administration and lower breast cancer incidence/recurrence [176]. 

### 5.3. Natural Compounds

A majority of therapeutic regimes rely on anticancer agents/compounds that, most of the time, have very specific targets. These therapies seem to work initially; however, as mentioned in the very beginning of this paper, a lot of such therapies become useless with the passage of time. Targeted drugs take care of their intended targets very effectively but, with the continued administration, tumor cells reduce their dependency on the targeted factor/pathways and switch to an alternative survival pathway. It has, therefore, been advocated that multitargeted cancer therapy should result in prolonged disease-free survival. Multitargeted therapy has its own shortcomings. Most of the targeted therapies come with their own associated toxicities. Throw in a mixture of such agents and we can end up with enormous side effects. These observations have come in the way of more detailed clinical studies aimed at combining anticancer drugs. One alternative is the use of natural agents, the compounds that exist in nature as constituents of various edible fruits/vegetables. A number of such compounds are pleiotropic that is, they target multiple signaling pathways [177–181]. Additionally, being part of normal human diet, they usually have considerably reduced toxic effects, when compared to conventional therapeutics. 

As direct evidence connecting natural anticancer compounds to reducing breast cancer recurrence, soy isoflavone consumption has been linked to reduced risk of breast cancer incidence and, more importantly, to the reduced risk of breast cancer recurrence [182]. CSCs are very crucial to the phenomenon of tumor recurrence, and several natural anticancer agents such as curcumin, sulforaphane, isoflavones, EGCG, and resveratrol have been shown to affect signaling pathways like Notch, Wnt, and Shh leading to their action against CSCs [183]. EGCG was shown to inhibit Wnt signaling through a regulation of HBP1 transcriptional repressor [184] and knockdown of HBP1 was found to result in reduced sensitivity to EGCG treatment.

The previous discussion highlights the connection between CSCs, EMT, and the various signaling pathways such as Notch, Wnt, and Hh that might influence breast cancer recurrence. A number of studies have described a beneficial effect of natural compounds through the modulation of these factors [181, 185–190], but a detailed discussion on these topics is beyond the scope of this paper and readers are encouraged to refer to the referenced articles. The recent literature has added miRNAs to the list of factors that may influence breast cancer recurrence, and there is evidence in the literature to suggest a regulatory effect of natural compounds on miRNAs as well [191–194]. 

## 6. Conclusions and Perspective

**Figure 3:**
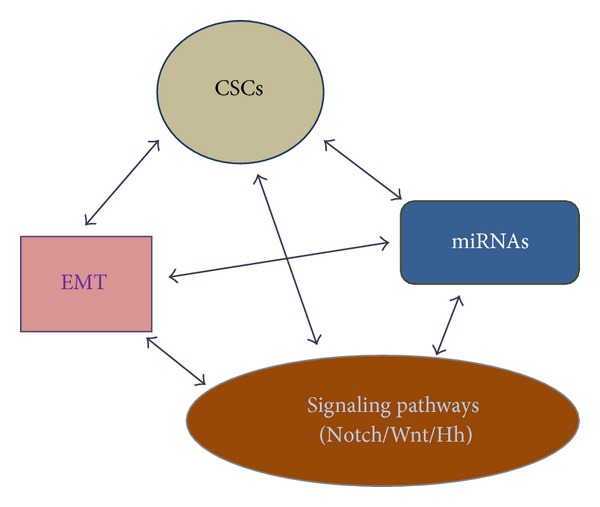
Complex interplay of factors that influence breast cancer recurrence. The various molecular determinants of tumor recurrence are very intricately connected and interregulated, making it difficult to establish a hierarchical sense.

The cause(s) of breast cancer recurrence and the possible strategies to prevent it remain elusive. There is an urgent need to decipher the complex relationship between individual components leading to recurrence but also to then come up with effective therapies with minimal toxicity. Talking about complex interrelationships, there is enough evidence to link EMT with Notch/Wnt/Hh signaling [112, 183, 194, 195], EMT with CSCs [51, 52, 187], EMT with miRNAs [73, 194, 196], Notch/Wnt/Hh signaling with CSCs [99, 105, 119, 177, 183], and CSCs with miRNAs [193, 196]. All this points to a hotchpotch of cellular/physiological events that have been observed in breast cancers with recurrence (Figure 3). While individual reports have verified the existence of one or more of these, it is essential to point out that we are far from ascertaining the causative reason for breast cancer recurrence. The hierarchy of these factors is not at all understood. With the intricate relationship between these individual factors, it will always be difficult to guess what happens first, a breast cancer recurrence equivalent of “chicken or the egg.” 

 Breast cancer mortality is largely related to either resistance to therapies or metastases to distant organs, all of which contribute to recurrence. One factor that has greatly hampered our progress in the field is the absence of any acceptable model for the study of tumor recurrence [197]. The *in vitro* studies are so often not “physiologically relevant” while the *in vivo* clinical observations are either so variable or simply inconclusive. For a meaningful study, it is vital to foster a healthy collaboration between the basic scientists and clinical investigators. Breast cancer recurrence is too complex a problem to be understood entirely through laboratory investigations or the clinical observations alone. The wealth of literature that has accumulated in the past several years is a good start. Now is the time for a concerted effort in order to provide a direction to the ongoing investigations through more meaningful, exhaustive collaborative projects that culminate in well-designed clinical trials. This alone can change the lives of scores of women suffering with recurrent, aggressive breast cancers with uncertain future, at least for the time being.

## Figures and Tables

**Table 1:** Evidence supporting a role of EMT markers in breast tumor recurrence.

EMT marker	Expression in recurrent breast tumors	Reference
E-Cadherin	Low	[59, 69]
N-Cadherin	High	[59]
Slug	High	[69]
Snail	High	[7, 58, 59]
Twist	High	[59, 61]
Vimentin	High	[69]

**Table 2:** miRNAs that influence breast tumor recurrence.

miRNA	Status in recurrent breast tumors	Reference
miR-9	Upregulated	[144]
miR-21	Upregulated	[147]
miR-30a	Downregulated	[150]
miR-31	Upregulated	[140]
miR-34a	Downregulated	[145]
miR-34c	Upregulated	[140]
miR-92a	Downregulated	[143]
miR-122	Upregulated	[151]
miR-125b	Upregulated	[152]
miR-126	Downregulated	[139]
miR-181a	Upregulated	[147]
miR-320	Downregulated	[146]
miR-335	Downregulated	[139, 141]
miR-451	Downregulated	[148]
